# Contextual Recalcitrance of Polylactic Acid (PLA) in Soils: Degradation Mechanisms, Microbial Traits, and Fate-Relevant Endpoints

**DOI:** 10.3390/jox16060183

**Published:** 2026-09-25

**Authors:** Jacob W. Clements, Michael L. McKinney, Jennifer DeBruyn, Douglas G. Hayes, Sean M. Schaeffer

**Affiliations:** 1 Department of Biosystems Engineering and Soil Science, University of Tennessee-Knoxville, Knoxville, TN 37996, USA; jdebruyn@utk.edu (J.D.); dhayes1@utk.edu (D.G.H.); sschaef5@utk.edu (S.M.S.); 2 Department of Earth, Environmental, and Planetary Sciences, College of Arts and Sciences, University of Tennessee-Knoxville, 1621 Cumberland Ave Rm 602, Knoxville, TN 37916, USA

**Keywords:** polylactic acid (PLA), soil biodegradation, contextual recalcitrance, microplastics, environmental persistence, microbial depolymerization

## Abstract

Polylactic acid (PLA) is among the most widely used biodegradable, biobased polymers, yet it can persist in soils and waters, where accumulation may affect plants and agroecosystems. This narrative, mechanistic review asks why PLA behaves differently under industrial composting and ambient soil conditions, drawing on original studies and using soils as the interpretive lens, with aquatic and waste management systems for contrast. We examine hydrolysis, Norrish photochemistry, biofilm formation, enzymatic cleavage, environmental controls, polymer crystallinity, physical accessibility, and microbial community interactions. Elevated temperature and moisture promote rapid degradation during industrial composting, whereas ambient conditions slow hydrolysis and restrict microbial access. We propose contextual recalcitrance as an organizing framework, in which persistence arises from interactions among polymer structure, environmental conditions, physical accessibility, and the local availability of depolymerizing traits rather than intrinsic chemical resistance. Within it, we advance trait gating by primary depolymerizers and cross-feeding of soluble lactate as a testable conceptual model rather than a demonstrated soil succession because community-resolved field evidence is lacking. We conclude that environmental biodegradability should be assessed using endpoints beyond CO_2_ evolution, including dissolved organic carbon, microbial assimilation, and organic matter partitioning, to distinguish fragmentation, depolymerization, assimilation, mineralization, and stabilization of PLA-derived carbon in soils.

## 1. Introduction

Biodegradable plastics are increasingly deployed as environmentally sustainable alternatives to conventional polymers in applications where recovery and recycling are structurally limited, including food service packaging, contaminated flexible materials, and agricultural mulch films. Their environmental fate, however, cannot be inferred from just the label “biodegradable”. Biodegradation is an outcome that depends on polymer chemistry, additives, supramolecular structure, and environmental context, including temperature, water availability, and microbial function [1]. This contextual dependence is especially relevant because, when dedicated collection and industrial composting are unavailable or unsuccessful, biodegradable plastic products may enter ambient soils, freshwater systems, marine waters, wastewater infrastructure, or landfills. The relative contribution of these pathways has not been quantified specifically for PLA; accordingly, we treat them as possible exposure routes rather than established dominant destinations.

Polylactic acid (PLA) is one of the highest volume compostable polymers and a dominant material in several major product categories [2]. In 2025, PLA accounted for 26.4% of the estimated 2.31 million tonnes of global biobased plastics production capacity, equivalent to approximately 0.61 million tonnes of installed annual capacity; this estimate describes production capacity rather than actual production, demand, or consumption [3]. Polylactic acid is also present in some agricultural biodegradable products, although it is not the primary polymer in most soil biodegradable mulch films. Commercial mulch films often consist of polybutylene adipate terephthalate (PBAT), polybutylene succinate (PBS), polylactic acid (PLA), polyhydroxyalkanoate (PHA), and additives to tune performance and biodegradation [4].

Soils can receive PLA through several documented and plausible pathways. Documented agricultural sources include PLA-containing products deliberately placed in or on soil, including some blended films and other plastic products (e.g., pots, trays, and crop supports). Based on pathways documented for other biodegradable plastics, we can also assume that PLA enters soils via land applications and mismanaged wastes. Compost and organic waste amendments may deliver incompletely disintegrated fragments of compostable packaging. Wastewater treatment concentrates microplastics as a class in sludge, but the contribution of PLA specifically to land-applied biosolids has not been quantified. Mismanaged consumer waste may also deliver PLA to soils through runoff, erosion, and redistribution throughout the soil–water interface [1,4].

PLA is also widely used outside agriculture, and many of the dominant waste streams that generate environmental release originate in packaging, consumer goods, and industrial applications. Packaging has historically been one of the largest application areas for PLA because it can be processed into films, rigid containers, and multilayer structures, and it is marketed as a compostable alternative to petroleum-based conventional packaging [5,6]. PLA is also used in biomedical and consumer products, and its applications continue to expand as formulations are tailored for stiffness, transparency, and printability [6,7]. Recently, a highly visible use of PLA filaments is fused deposition modeling-based 3D printing, creating a distributed waste stream that includes failed prints, support structures, and sanding debris that can enter municipal waste streams or be mishandled as litter [8].

These non-agricultural uses may connect to environmental compartments through several transport pathways. Littering and mismanaged solid waste can deliver PLA items directly to surface waters or terrestrial environments, where they may fragment and persist under mesophilic conditions. Wastewater treatment plants receive microplastics from household and commercial sources, retain much of that material in sludge, and discharge a fraction in effluent to aquatic systems [9,10]. However, these studies address total microplastics and do not resolve PLA-specific occurrence or flux. Because PLA is present in consumer products, fragmented debris or fibers could enter sewer systems, and sludge or biosolids application could transport residual PLA to soils; at present, this should be regarded as a plausible pathway rather than a demonstrated major PLA flux.

In this review, we treat PLA as a case study to understand biodegradable plastic fates in the environment. This requires citing literature from the fields of polymer chemistry, microbial ecology, and soil science. Existing reviews (e.g., [11,12,13,14]) synthesize PLA degradation from polymer/materials, environmental compartment, or soil/biodegradable mulch perspectives; Hajilou et al. [14], for example, compared soil, compost, water, and wastewater and emphasized mathematical modeling. Our distinct contribution is to integrate polymer structure, environmental conditions, physical accessibility, and the local availability of depolymerizing traits within the framework of contextual recalcitrance, then connect those controls to microbial carbon allocation and fate-relevant endpoints. While we primarily focus on soils, we recognize that related transformation mechanisms operate in aquatic and waste management contexts. We review mechanisms that influence polymer breakdown and downstream carbon processing, then interpret variability through a trait-based microbial ecology framework. In soils, carbon turnover often depends on physicochemical access, extracellular transformation, and microbial allocation, not simply the presence of degraders [15,16,17]. We define contextual recalcitrance as persistence arising from interactions among polymer structure, environmental conditions, physical accessibility, and the local availability of depolymerizing traits, rather than from intrinsic chemical resistance alone. We propose trait gating, primary depolymerizers, secondary consumption, and cross-feeding as a testable conceptual framework where direct community-resolved field evidence is lacking. We build on this framework to distinguish fragmentation, depolymerization, assimilation, mineralization, and possible retention in soil carbon pools, clarify when compostability claims are consistent with environmental fate, and connect incomplete transformation to PLA-specific ecotoxicity evidence. Rather than focusing on materials optimization, we emphasize fate-relevant endpoints to improve environmental benchmarking of PLA biodegradability.

## 2. Methods

We conducted a targeted narrative and mechanistic literature search to review evidence for PLA occurrence, transformation, and biodegradation mechanisms across environments, using soils as the primary interpretive lens. This was not designed as a systematic review or meta-analysis. We also contrasted field and mesocosm outcomes with standardized biodegradability testing frameworks used for compostability, soil, and water claims. Searches were performed in Web of Science, Scopus, and Google Scholar from October 2025 to August 2026 using combinations of “polylactic acid” or “PLA” with terms capturing environmental compartments and processes, including “soil,” “freshwater,” “river,” “lake,” “marine,” “seawater,” “wastewater,” “biosolids,” “sludge,” “landfill,” “leachate,” “compost,” “industrial compost,” and “home compost.” Mechanistic terms included “hydrolysis,” “depolymerization,” “ester cleavage,” “molecular weight,” “crystallinity,” “glass transition,” “biofilm,” “cutinase,” “lipase,” “esterase,” “depolymerase,” and “proteinase,” along with “microplastic,” “fragment*,” and “surface erosion.” Standards and test method terms included “ASTM,” “ISO,” and specific frameworks such as “incubations” relevant to composting and environmental biodegradation testing. Title and abstract screening was followed by full-text assessment and backward citation searching of included papers.

We prioritized English-language, peer-reviewed original studies conducted under environmentally realistic, mesophilic conditions in soil and aquatic settings when they reported at least one endpoint relevant to polymer transformation or carbon fate, including CO_2_ evolution, mass or surface area loss, molecular weight change, fragment abundance, dissolved organic carbon or lactic acid release, microbial biomass incorporation, or association with particulate and mineral soil fractions. Studies of PLA blends were included when PLA was an identifiable component and the measured outcome could be interpreted for the PLA-containing formulation. Optimized composting tests were included as benchmarks for interpreting differences between certification environments and ambient compartments, not as direct proxies for in situ behavior. We excluded studies concerned only with polymer synthesis or mechanical performance, studies of other polymers without a PLA comparator, and studies that inferred biodegradation solely from visual change or mass loss without acknowledging that these endpoints can reflect disintegration or abiotic transformation. Foundational soil carbon and microbial trait literature was included only to interpret substrate accessibility, physical protection, extracellular enzyme constraints, and community-level processing of hydrolysis products. Reviews were used for broad context and citation chaining, while PLA-specific quantitative values and mechanistic claims were checked against original articles where available. To ground the discussion in empirical outcomes, Table S1 summarizes peer-reviewed original studies reporting PLA transformation and biodegradation endpoints in soil, soil-like, and aquatic systems [18,19,20,21,22,23,24,25,26,27,28,29,30].

Evidence was evaluated by the process directly measured rather than by a numerical quality score. We treated PLA-specific field or soil/aquatic microcosm studies with isotope attribution or carbon mass balance as the strongest evidence for environmental carbon fate, followed by PLA-specific microcosm studies with polymer transformation endpoints, isolate or whole-cell studies, purified enzyme studies, and indirect inference from the non-PLA soil carbon literature. Conflicting results were not averaged across incompatible conditions. Instead, they were interpreted according to the evidence categories in Table 1, the endpoint hierarchy in Table 2, and the reported temperature, moisture, medium, particle size, molecular weight, crystallinity, formulation, and exposure duration.

### Scope and Limitations of the Evidence Base

This article is a targeted narrative and mechanistic review rather than a systematic review or meta-analysis, and its scope should be read accordingly. Searching was iterative and relied on database relevance ranking and citation chaining rather than a prospectively registered protocol, so results were not exported into a single fixed corpus, and formal retrieval, duplicate removal, and exclusion counts were not generated; we state this explicitly rather than imply PRISMA-style coverage. The heterogeneity in the PLA literature, especially differences in temperature, moisture, medium, particle size, molecular weight, crystallinity, formulation, and exposure duration, also precluded a quantitative meta-analysis and is the reason this synthesis is organized around mechanisms and evidence categories rather than pooled effect sizes. Where PLA-specific evidence is absent, we mark the corresponding statements as hypotheses or as conceptual frameworks rather than as findings, and Table 1 records the evidence category supporting each mechanistic claim.

## 3. The Concept of Recalcitrance: Soil Organic Matter and Added Substrates

Soil is a significant reservoir of carbon on Earth, containing organic carbon in various forms that differ in persistence. When plant residues or other organic inputs, such as biodegradable plastics, enter the soil, they decompose along multiple pathways. Some carbon is rapidly catabolized by microbes into CO_2_, while the remainder is transformed into soil organic matter that can be conceptualized as pools defined by stability and turnover time. Key pools include fresh residues, particulate organic matter (POM), mineral-associated organic matter (MAOM), dissolved organic matter (DOM), living microbial biomass, and microbial necromass, which together span a continuum from labile, rapidly cycled forms to highly stabilized forms protected from decomposition by mineral association. Carbon stabilization occurs through multiple pathways, including organo-mineral associations and aggregation with other SOC particles or inside microbial biomass [17]. Because substrates differ in molecular size, solubility, and structural complexity, they can require different degrees of extracellular transformation before becoming broadly assimilable by microbes. Glucose and lignin are often considered conceptual endpoints along an accessibility/processing continuum [17]. Figure 1 illustrates how accessibility and processing requirements regulate the biological availability of PLA carbon compared to other common substrates (Figure 1A) and the potential pools into which that carbon may subsequently be partitioned (Figure 1B, [17]).

MAOM often represents a relatively persistent SOC pool because sorption to clay and oxide surfaces, co-precipitation with metal ions (e.g., Fe^2+^/Fe^3+^ and Al^3+^), and physical separation within microaggregates can reduce accessibility to enzymes. Reported residence times can span decades to centuries at aggregate or modeled pool scales, but these are not fixed properties of MAOM; persistence varies with mineralogy, climate, the type and strength of organo-mineral associations, mineral saturation, management, and soil disturbance [16,37,38,39]. Frameworks such as Microbial Efficiency–Matrix Stabilization (MEMS) emphasize that microbially processed carbon, especially necromass, can contribute to persistent organo-mineral complexes [16], and continuum-based models point out that persistence is governed more by environmental and structural protection than by intrinsic chemical recalcitrance [17,38]. Recent work further refines how mineralogy, climate, and management control stabilized pool size and durability [39], supporting MAOM as a focal target for sequestration strategies [40]. In contrast, free POM, DOM, and microbial biomass generally turn over more rapidly, while POM may persist longer when physically protected or transferred to MAOM [17,41].

## 4. PLA as a Substrate

For PLA, polymer traits set the baseline for biodegradation, and environmental controls modulate it [42]. PLA’s ester linkages make it hydrolysable, but stereochemistry (how the atoms arrange in space by using different chiral monomer units—L-lactide and D-lactide—which twist light differently and create distinct crystal structures and melting points, in this case: poly-L-lactic acid; PLLA vs. poly-D-lactic acid; PDLA vs. poly-D-L-lactic acid; PDLLA) and crystallinity govern chain mobility and enzyme access. PLA stereochemistry strongly controls its degradation because the L/D configuration governs crystallinity and stereocomplex formation, which in turn regulate water uptake, ester bond accessibility, and enzyme stereoselectivity [43]. Isotactic or stereocomplex PLA forms tightly packed crystallites that hydrolyze and mineralize more slowly than amorphous, racemic PLA, changing overall degradation rates. Amorphous domains hydrolyze first, but crystalline regions persist and can even grow as amorphous regions erode, raising apparent recalcitrance at mesophilic temperatures [12,44]. Soil microbial enzymes, such as cutinases, lipases, and protease-like serine hydrolases, act at the surface. Therefore, it follows that values of water activity, pH, and temperature that promote ester hydrolysis and swelling also raise rates.

In conditions below the glass transition temperature (T_g_), which is the temperature where the plastic transitions from a rigid solid into a soft rubbery material, and at low moisture, depolymerization is diffusion-limited [45]. Field soils are mesophilic and mineral-rich, with variable pH and moisture. In contrast, industrial composting provides sustained high temperature and moisture, so PLA degrades quickly once T_g_ is exceeded. Consequently, rates of degradation under composting conditions do not automatically translate to field soils [44,46]. These polymer-by-context interactions explain why PLA appears recalcitrant in field conditions yet is readily mineralized in compost.

In addition to environmental conditions, formulation can affect degradation, but these effects are not uniform. In controlled PLA systems, thermoplastic starch increased hydrophilicity, reduced hydrolysis lag, and increased mineralization under composting conditions, while montmorillonite nanoclays shortened the lag phase and accelerated hydrolytic chain scission; blends with PBSA or PCL also increased biodegradation at mild composting temperatures [47,48,49]. These results are specific to the tested formulations and conditions. Plasticizers, CaCO_3_, pigments, and nucleating agents can alter morphology, crystallinity, and water uptake; therefore, the direction and magnitude of any change in PLA transformation must be demonstrated for each formulation rather than generalized from additive class [12].

## 5. Mechanisms and Environmental Controls of PLA Transformation in Soil

### 5.1. Chemical Structure and Abiotic Depolymerization

Polylactic acid is an aliphatic polyester whose backbone consists of repeating lactyl units joined by ester linkages. Hydrolysis and transesterification cleave these ester linkages [50,51], while photochemical pathways generate radical-mediated chain scission following excitation of carbonyl groups [52,53,54]. Each hydrolytic ester scission event creates new hydroxyl and carboxyl termini. Since an increase in carboxylic acids can catalyze further ester hydrolysis, hydrolytic degradation can become autocatalytic. In sufficiently thick samples, it may accelerate when acidic oligomers accumulate faster than they diffuse outward, producing bulk erosion rather than purely surface-limited loss [51]. Chain-end processes contribute to abiotic depolymerization as well. The terminal hydroxyl group can attack nearby ester bonds intramolecularly and release cyclic oligomers and lactide by back-biting, so molecular weight loss reflects both random scission along the backbone and end-group-mediated depolymerization [50]. Continued cleavage then yields water-soluble oligomers and ultimately lactic acid, the monomer available for microbial uptake.

These routes differ in what initiates attack, and the distinction matters for interpreting environmental data. At acidic pH, PLA hydrolysis often shows preferential chain-end scission, whereas neutral or alkaline conditions can promote backbiting and random ester cleavage. The relative contributions of these pathways depend on polymer molecular weight, morphology, and reaction conditions [50,51]. Thermal and photochemical routes can also initiate chain scission through different mechanisms. Elevated temperature accelerates hydrolysis and permits non-hydrolytic transesterification, while ultraviolet exposure generates radicals through Norrish chemistry. Enzymatic cleavage is chemically the same as ester hydrolysis but is restricted to the solid surface and requires an active site that accommodates the lactyl unit. Under ambient soil temperature and pH, purely abiotic routes are slow, which is why crystallinity, water access, and the local availability of compatible enzymes exert more control over PLA persistence than the intrinsic reactivity of the ester bond [12,20,24,42,55].

### 5.2. Physical Accessibility and Surface Conditioning

Soil imposes several physical constraints on microbial and enzymatic access to PLA. A key factor is particle size. Small particles (e.g., microplastics) offer substantially more surface area per mass for colonization and attack relative to large particles or films [12]. Weathering processes such as UV exposure, thermal cycling, and abrasion can induce surface erosion and microcrack formation in PLA materials, which in turn increases their surface area and therefore bioavailability. Under UV exposure specifically, PLA can undergo carbonyl-mediated photolysis via Norrish Type I (α-cleavage) and Norrish Type II (γ-H abstraction) pathways, producing radical formation, chain scission, and oxidized end groups that lower molecular weight and embrittle the surface [52,53]. These photochemical changes can amplify mechanical cracking and surface roughening, which promotes water ingress and increases the number of interfacial sites available for microbial attachment and extracellular enzymes [42,54].

PLA transformation commonly involves interacting physicochemical and biological processes, including water ingress, photochemical and mechanical weathering, surface fragmentation, microbial colonization, and extracellular enzyme attack. These processes can occur concurrently and iteratively, increasing polymer accessibility before or alongside substantial microbial mineralization [12]. Biofilm colonization is frequently observed during early degradation and helps retain moisture, cells, and extracellular enzymes at the polymer interface (Figure 2, [11,56]). Biofilms facilitate depolymerization of PLA’s ester bonds. However, biofilms can also act as diffusional barriers. High cell density and extracellular polymeric substances may locally deplete oxygen or slow the release of soluble by-products. Overall, initial surface conditioning by forming cracks, pits, and biofilm development is critical for enabling PLA to be more accessible to the wider soil microbiome [33,42].

Beyond the polymer’s own surface characteristics, the soil matrix plays a role in PLA bioavailability. PLA fragments can become occluded within soil aggregates or trapped in pores, physically shielding them from contact with microbes and extracellular enzymes. In their soil continuum model, Lehmann and Kleber [17] emphasize that the spatial layout of organic matter, whether free in soil or protected in aggregates, strongly influences decomposition rates by controlling microbial access. Consequently, a PLA particle inside a stable soil microaggregate may persist much longer due to the physical exclusion of decomposers. Additionally, mineral association is a possible but untested fate for PLA breakdown products. Soil minerals adsorb many low-molecular-weight organic compounds [17], but direct measurements showing that lactate or PLA oligomers released during PLA transformation bind to clay or oxide surfaces and thereby reduce microbial uptake are currently lacking. We therefore treat mineral association as a testable hypothesis rather than an established contributor to PLA recalcitrance. If sorption occurs, it could transiently partition soluble products to mineral surfaces and alter their immediate availability and downstream fate; this requires validation using labeled PLA products and mineral-specific sorption assays. This means that even though PLA is chemically susceptible to hydrolysis, its degradation in soil is constrained by physical factors that govern the accessibility of the polymer to the organisms and enzymes that catalyze its breakdown.

### 5.3. Enzymatic Depolymerization

Extracellular microbial depolymerization represents a critical biological bottleneck because intact high-molecular-weight PLA cannot be directly transported into microbial cells [24]. A variety of extracellular enzymes can cleave the ester linkages in PLA, including cutinases, esterases, lipases, and certain proteases [31,34,35,42,57]. These enzymes, often secreted by specialized bacteria and fungi for other purposes, hydrolyze PLA into smaller oligomers and lactic acid monomers [45]. Cutinases, which are enzymes that originally target plant cutin polyesters, have shown particularly high activity on aliphatic polyesters like PLA, whereas lipases and esterases (which typically act on lipids or simple esters, respectively) also contribute to PLA degradation in various microbes. Importantly, PLA-degrading enzymes typically function at the solid–substrate interface. Microbes must attach or remain near PLA to efficiently secrete depolymerases and assimilate the released products. Only after abiotic or enzyme-mediated scission reduces the polymer to water-soluble oligomers and monomers can microbes transport and metabolize the carbon.

### 5.4. Uptake of Soluble Products and Mineralization

Once extracellular enzymes have broken down PLA into soluble oligomers and monomers, these smaller products become available for microbial uptake. The primary monomer released is lactic (2-hydroxypropionic) acid, which is readily utilized by many soil microbes. At neutral pH (pKa = 3.86), lactic acid occurs predominantly as lactate and can move through soil water films. Cellular uptake is transporter-mediated; characterized microbial systems include lactate: H^+^ symporters and other cation-coupled 2-hydroxycarboxylate transporters or exchangers, which generally use electrochemical gradients rather than direct ATP hydrolysis [58].

Tracing carbon past the point of uptake completes the mineralization sequence. Inside the cell, lactate is oxidized to pyruvate by lactate dehydrogenase, and pyruvate is either fully oxidized through the tricarboxylic acid cycle to CO_2_ or routed into anabolic pathways, as developed in Section 6 [42,59]. Complete mineralization under oxic conditions therefore requires the entire sequence of surface conditioning, extracellular depolymerization, transport, and intracellular oxidation, and a constraint at any one step limits the CO_2_ yield that a biodegradation test records. The terminal product of PLA-derived carbon is set by the prevailing electron-accepting conditions rather than by the polymer alone, and mineralization endpoints should specify which terminal product was measured.

### 5.5. Environmental Controls on Transformation Rates

Environmental factors, such as temperature, soil moisture, and pH, influence the activity of PLA-degrading enzymes. Temperature is a well-known factor that controls enzymatic degradation. Enzymatic PLA breakdown accelerates with increasing temperature, and many PLA-depolymerizing enzymes that have been identified to date have optimal activity in the thermophilic range [12,31,60]. Under industrial composting conditions, which commonly reach 55–70 °C, PLA can undergo substantial biodegradation and mineralization over periods of months, depending on formulation and test conditions. This is partly because enzymes like cutinases operate near their optimal temperature of 50–60 °C, helping the polymer chains undergo faster hydrolysis. By contrast, in mesophilic soils (~10–30 °C), enzyme activity is much lower, and PLA degradation rates drop accordingly [24,42]. Soil moisture is another key factor. Hydrolytic enzymes require water for their reaction, and the diffusion of enzymes and monomeric products is water-dependent. Drought conditions drastically slow PLA depolymerization, reducing hydrolysis reactions due to insufficient water for enzyme function and for polymer matrix swelling. Additionally, pH conditions modulate both enzyme stability and abiotic hydrolysis rates. Most PLA-degrading enzymes from soil microbes have optimal activity in neutral and slightly alkaline pH. Consequently, if the soil is very acidic or basic, enzyme activity can be inhibited. High pH can catalyze abiotic hydrolysis of PLA’s ester bonds; however, in typical soils (pH ~5–8), abiotic hydrolysis of PLA is extremely slow, so biological catalysis remains the dominant breakdown pathway [20,24,42]. In laboratory soil incubations under ambient temperatures and neutral pH, PLA without additives or blend components showed negligible mass loss unless active microbes are present [12,21,24,26]. However, when the soil was bioaugmented with a known PLA-degrading microbial strain, the rate of PLA breakdown increased significantly [24]. Under mesophilic soil conditions, limited biological depolymerization appears to be a dominant constraint, although it interacts with slow abiotic hydrolysis, polymer structure, moisture availability, and physical accessibility.

## 6. Functional Guilds Involved in PLA Degradation

In soil, the assimilation and utilization of PLA-derived monomers can involve many different microbes. We use “primary depolymerizer” for an organism that colonizes PLA and secretes compatible extracellular enzymes and “secondary consumer” for organisms that use released oligomers or lactate without necessarily attacking intact PLA. This is a conceptual framework rather than a demonstrated succession in soils. A primary depolymerizer may also consume the products it releases, and direct community-resolved field evidence of cross-feeding remains lacking. PLA-specific soil microcosms show that PLA hydrolysate can be preferentially assimilated by Gram-negative bacteria and that different bacterial groups can contribute differently to PLA-derived CO_2_ [36]. Satti et al. [24] also noted that adding a PLA-degrading strain to soil increased total microbial activity, which is consistent with, but does not directly demonstrate, downstream consumption by other community members. Soil microbes with stereospecific L- and D-lactate dehydrogenases can oxidize lactate to pyruvate, which then enters respiratory or anabolic pathways; under anoxic conditions, further products depend on available electron acceptors. These observations support testing, rather than assuming, how PLA stereochemistry and community composition influence the taxa that take up PLA-derived carbon.

PLA-derived carbon can enter microbial biomass through direct use by primary depolymerizers and through secondary cross-feeding of released lactate and oligomers. The dominant mode likely depends on the organism and environmental context. In some specialist taxa, extracellular depolymerase production appears growth-linked because PLA can serve as the sole carbon source, providing a direct metabolic energy return on enzyme investment [31]. Other work on PLA-degrading Actinobacteria indicates substantial strain-level variation in PLA colonization traits, including biofilm formation, lactic acid assimilation, and constitutive versus condition-dependent depolymerization activity [33]. At the same time, co-metabolic depolymerization is plausible in soil because many enzymes reported to attack PLA belong to a diverse hydrolase family that includes enzymes involved in catabolism of other substrates. The production of these enzymes by microbes may result in PLA cleavage, even when the microbes’ growth is supported by alternative carbon inputs [45,61]. These two strategies imply different carbon fate expectations. When depolymerization is growth-linked, the primary degrader may catalyze the conversion of a larger share of PLA-derived carbon into biomass. When depolymerization is co-metabolic, released lactate and oligomers are more likely to be rapidly scavenged by the surrounding community, increasing cross-feeding and weakening any one-to-one correspondence between the identity of depolymerizers and the taxa that assimilate PLA-derived carbon.

One consequence of competition between primary degraders and secondary consumers is that PLA degradation may not strongly enrich the growth and activity of primary depolymerizers unless it provides a competitive advantage in obtaining the hydrolysis products. If lactate is rapidly scavenged, the organisms paying the extracellular enzyme cost can experience weaker energetic returns. At the community level, however, fast consumption of soluble products can still accelerate overall carbon turnover by maintaining a concentration gradient away from the polymer surface while decreasing local product build-up, which effectively couples slow depolymerization to rapid downstream metabolism. In soils, low-molecular-weight organic acids are typically short-lived in solution because microbial uptake is extremely rapid and often out-competes sorption processes, so dissolved pools can remain low and difficult to detect even when production rates are substantial [62,63]. This expectation is consistent with observations that low-molecular-weight organic acids are commonly present at low concentrations in soil solutions and are quickly processed [64], and with experiments showing preferential microbial use and rapid removal of organic acids in intact soils. Because lactate is readily metabolized across soil and sediment communities, and because enantioselectivity can influence uptake dynamics, lactate stereochemistry may further influence which taxa dominate consumption [65]. These general organic acid observations motivate the hypothesis that lactate released from PLA will often be rapidly metabolized, but the rate and identity of consumers must be measured directly in PLA-amended soils. Low lactate concentrations therefore cannot by themselves distinguish slow production from rapid uptake or demonstrate cross-feeding.

The fate of lactate in microbial metabolism is not uniform. Different microbes may channel it into different metabolic pathways, with some taxa fully oxidizing it and others incorporating it into biomass, leading to a distribution of PLA-derived carbon across the microbial community. Once inside microbial cells, lactic acid enters central metabolic pathways. Typically, lactate is oxidized to pyruvate by lactate dehydrogenase or a related oxidase. Pyruvate, a key metabolite, can be routed into the tricarboxylic acid (TCA) cycle for complete oxidation to CO_2_ and energy production or diverted into anabolic pathways to form building blocks for new microbial biomass. In aerobic soil microbes, pyruvate usually enters the TCA cycle, yielding reducing power (NADH) and eventually ATP via oxidative phosphorylation. Therefore, carbon from PLA can be fully oxidized to CO_2_ and water or transformed through anabolism into oxaloacetate or acetyl-CoA to synthesize amino acids, lipids, and sugars for cell growth [59]. Qi et al. [42] noted that microorganisms ultimately assimilate PLA’s carbon either into biomass or mineralize it to CO_2_ depending on their metabolic demands. The partitioning of assimilated PLA-derived carbon between respiration and microbial growth depends on microbial carbon use efficiency (CUE), defined here as the proportion of assimilated carbon allocated to biomass rather than respired as CO_2_. This is relevant to PLA environmental fate because carbon incorporated into microbial biomass could subsequently enter microbial necromass and longer-lived soil organic matter pools, whereas respired carbon is lost to the atmosphere. CUE therefore provides a conceptual link between PLA depolymerization, microbial assimilation, and the downstream partitioning of PLA-derived carbon among CO_2_, biomass, POM, and MAOM [16,17,59].

## 7. Functional Trait Framing of PLA Degraders

One way that microbial ecologists characterize decomposer organisms is by trait continua, linking them to the carbon substrates they target. A key axis of a trait continuum is substrate recalcitrance, ranging from simple, labile compounds like glucose to complex, recalcitrant polymers like lignin. This continuum is reflected in bond structure and microbial acquisition strategies. Glucose, a monosaccharide with easily metabolized C–C and C–H bonds, can be directly taken up by virtually any heterotrophic microbe, requiring no extracellular breakdown. By contrast, cellulose consists of sturdy β-1,4-glycosidic bonds forming fibrous, crystalline microfibrils. Cellulose is water-insoluble and must be depolymerized by secreted cellulases. Only filamentous fungi and certain specialized bacteria possess this trait, so cellulose persistence is longer relative to glucose. Lignin, a phenylpropanoid heteropolymer in plant cell walls, is highly recalcitrant. Lignin’s complex phenolic bonds and heterogeneous structure provide resistance to most conventional hydrolases, requiring oxidative enzymes produced primarily by select fungi and some *Actinobacteria*. Consequently, lignin decomposes very slowly, and only a narrow guild of organisms can initiate its decay. Empirical studies show that such substrate complexity strongly influences microbial guild structure and activity. For instance, labile carbon additions rapidly stimulate fast-growing generalists, whereas only specialized slower-growing taxa respond to complex polymers [66,67] (Table 1).

^13^C stable isotope probing (SIP) can also show that the bioavailability of a carbon source explains some of the variation in bacterial assimilation, grouping soil taxa into distinct guilds based on whether they consume simple versus complex substrates [67]. Trait trade-offs therefore underlie these patterns. Microbes that invest in costly extracellular depolymerases often have slower maximum growth rates or lower growth yields. Conversely, microbes that thrive on freely diffusible substrates are capable of allocating more towards rapid growth and replication [66]. This is consistent with classic copiotroph–oligotroph strategy distinctions, where copiotrophic bacteria excel at exploiting simple, abundant sugars, while oligotrophic and polymer-degrading organisms endure lower resource quality by using enzyme-driven foraging strategies [66,68].

Consequently, to understand how microbes degrade PLA, we must understand its recalcitrance. Although PLA’s ester bonds are hydrolysable, its solid polymer form and high molecular weight make it inaccessible to microbes without depolymerization. In terms of bond structure and the extent of enzyme depolymerization required, PLA resembles cellulose. The majority of soil microbes, which can readily consume simple sugars like glucose, most likely will not directly utilize intact PLA. Instead, a minority group of microbes must secrete esterase and cutinase-like enzymes to cleave the polymer into oligomers and monomers, namely, lactic acid, before assimilation can begin [69,70]. Certain soil microbes have demonstrated the ability to attack PLA’s ester bonds, notably filamentous *Actinomycete* bacteria such as species of *Amycolatopsis*, *Actinomadura*, and *Streptomyces* [14,32,33]. These organisms are also well-known decomposers of plant polymers, consistent with the idea that polymer degradation capacity clusters in specific taxa. Actinomycetes and some thermophilic *Bacillus* spp. can produce extracellular PLA-depolymerases that hydrolyze PLA, especially under warm conditions [60]. Culture-based screening surveys indicate that PLA-depolymerizing activity is concentrated among *Actinomycetes* in culture collections [32,33], although their prevalence in field soils has not been established; soil and compost incubations further show that PLA breakdown is markedly accelerated under thermophilic conditions [18,21].

PLA’s position on the recalcitrance spectrum (Figure 1) could be toward the resistant-polymer end with lignocellulose. We hypothesize that under mesophilic soil conditions, intact PLA may be less biologically accessible than cellulose when compatible depolymerizing traits are rarer, despite PLA’s hydrolysable ester bonds. Direct side-by-side comparisons under standardized soil conditions are currently insufficient to assign a universal ranking, so the relative position in Figure 1 is conceptual and context-dependent. PLA can therefore supply a carbon resource that is initially outside the metabolic reach of much of the community. This reversal across environments is the central example of contextual recalcitrance. Under thermophilic composting conditions, elevated temperature and moisture accelerate hydrolysis, and compatible microbial communities metabolize the resulting products, allowing PLA to mineralize over months [14,20,22]. PLA is therefore not intrinsically positioned at a single point on a recalcitrance spectrum; its apparent lability depends on environmental conditions and microbial traits.

Soil carbon flow is governed by the matchup between substrate type and the functional traits present in the community. PLA’s fate therefore depends partly on the local abundance of organisms with compatible colonization and depolymerization traits [71]. By analogy with plant litter decomposition, we propose a primary–secondary pattern as a testable model rather than an established sequence for PLA in soil: a slow depolymerization phase may be followed by mineralization, assimilation, or sorption of released lactate and oligomers. Under controlled composting conditions, PLA mineralization curves often show an induction period before CO_2_ evolution accelerates [47,72], but this pattern is only consistent with an upstream transformation step and does not by itself identify microbial guild succession. PLA primary depolymerizers would be microbes that colonize PLA surfaces and secrete compatible depolymerases. *Amycolatopsis* strains have been shown to erode PLA films and assimilate lactic acid [33], demonstrating that depolymerization and downstream monomer use can occur in the same organism and cautioning against a strict division of labor. Soil microcosms showing increased PLA transformation under bioaugmentation or biostimulation support microbial limitation [24], but direct taxa-resolved evidence for a sequential primary-depolymerizer/secondary-consumer pathway in ambient soil is still lacking.

Applying the primary–secondary framework developed for plant litter decomposers to xenobiotic degraders highlights a few nuances. One is the potential absence or delay of primary degraders for plastics in a given environment. Because PLA is an anthropogenic xenobiotic substrate, a soil with no history of PLA addition may initially lack microbes that express the needed enzymes in significant numbers, leading to longer lag times before appreciable degradation commences. Studies often report a lag phase in PLA biodegradation [14,21,24,33,48,49,73,74], but these curves do not establish whether the delay reflects abiotic conditioning, enzyme induction, community adaptation, or proliferation of rare specialists. In agricultural soil, PLA could therefore persist for extended periods, while changes in primary degrader abundance remain a testable hypothesis. Some PLA-degrading enzymes are essentially cutinases, hinting that PLA degradation in soil may often be carried out by organisms that originally target plant cuticle or other substrates [57]. Repeated exposure could select for organisms capable of colonization or depolymerization, but we retain this as a hypothesis because direct evidence that repeated PLA additions accelerate subsequent transformation in ambient soil remains limited. Targeted addition of agricultural microbes has altered soil microbial community composition and increased PLA film mass loss and molecular weight decline [75], demonstrating that the availability of compatible taxa can influence transformation without establishing an exposure history effect.

The distinction between primary and secondary degraders may blur as microbial interactions become complex. Some microbes may partially degrade PLA exoenzymatically and assimilate a portion of the released monomers or oligomers, having characteristics of primary and secondary degraders. Others may engage in cross-feeding. However, the overarching concept of a functional separation between those that initiate attack on the xenobiotic polymer and those that assimilate or further metabolize released monomers and oligomers remains useful. The distinction proposed by Morrissey et al. [71] between primary and secondary degrader guilds can thus be evaluated for PLA by treating polymer depolymerizers as an analogue to litter primary decomposers and heterotrophs utilizing released lactate as potential secondary consumers. The microbial trait axes (Figure 1) could also help predict PLA degradation dynamics. Organisms with traits such as high growth rates and preference for monomers will not act until monomers are present, whereas those with high enzyme production and polymer utilization are needed to start the process. Barnett et al. [67] showed that soil bacteria segregate into guilds based on carbon use, with some specializing in complex substrates and others in simple ones.

## 8. Plastisphere Colonization as a Trait Gate

Plastic debris supports surface-attached microbial consortia, often termed the plastisphere, that differ from free-living communities because the substrate imposes strong selection for attachment, biofilm formation, and utilization of scarce or slowly released carbon sources [76,77]. The soil plastisphere includes the thin zone of soil under immediate influence of plastic particles, where microbial assemblages on and near plastic can diverge from bulk soil and where colonization may influence both polymer transformation and localized carbon cycling [78]. Plastisphere communities can alter local microenvironments at the polymer surface through extracellular polymeric substances, redox and pH gradients, and enzyme localization, which can amplify the importance of surface traits relative to water processes [76,77]. These surface processes are relevant for PLA because depolymerization depends on water access, enzyme contact, and the formation of biofilms that maintain proximity between cells and the polymer surface.

For biodegradable plastics such as PLA, the plastisphere lens aligns with a trait-based framework because the earliest steps in depolymerization are likely gated by surface colonization traits, extracellular depolymerase production, and the ability to persist at the polymer interface under moisture and temperature constraints [78]. Practically, this implies that “who arrives first” and “who can maintain attachment and enzyme activity on the PLA surface” can influence subsequent cross-feeding dynamics and the partitioning of PLA-derived carbon between respiration and retained soil pools. We therefore define the PLA plastisphere trait gate as the combined requirement for successful surface colonization, persistence at the polymer–soil interface, and extracellular depolymerase expression before PLA-derived carbon becomes broadly available to the surrounding microbial community. Analogous priority effects are well established in plant litter and wood decomposition, where the identity and functional traits of early colonists can redirect subsequent community assembly and decomposition trajectories [79]. This provides an ecological analogue for the proposed PLA plastisphere trait gate, although priority and home-field-advantage effects have not yet been tested directly for PLA.

## 9. Fate of PLA-C: Role of CUE

CUE is a central factor in determining how much PLA-derived carbon is retained in soil microbial biomass, and potentially soil organic matter, versus lost to the atmosphere. When PLA depolymerization occurs, the fate of liberated lactate and oligomers depends on microbial metabolic allocation. High CUE conditions promote the conversion of PLA-derived carbon into microbial biomass and, over time, microbial necromass, which could contribute to longer-lived SOC pools, consistent with the idea that more utilized substrates can yield more microbial residues and thus contribute more to stable SOM formation [16,59]. Low CUE conditions promote respiration, resulting in greater CO_2_ release and less retention of carbon in the soil. Qi et al. [42] similarly emphasized that microorganisms can ultimately assimilate PLA’s carbon into biomass or mineralize it to CO_2_ depending on metabolic demands, which maps directly onto CUE.

However, PLA introduces a critical upstream constraint that is not present for readily soluble substrates such as glucose. For PLA, apparent community-level CUE therefore integrates both the energetic cost of extracellular depolymerization and the metabolic allocation of soluble products, potentially across different organisms. The initial depolymerization step often requires extracellular enzyme production and occurs slowly under mesophilic soil conditions, which can decouple polymer transformation from rapid microbial growth. In a community context, this can shift the relationship between depolymerization and CUE because the organisms investing in depolymerase production may not be the same organisms capturing the hydrolysis products. Cross-feeding can redistribute PLA-derived carbon across the community, carrying implications for both mineralization and stabilization. In soils, microbial processing and turnover often contribute disproportionately to MAOM formation, especially when microbial residues interact with mineral surfaces, so understanding the conditions that raise or lower CUE helps predict whether PLA-derived carbon is more likely to be retained as stabilized SOC or lost as CO_2_ [16,17,59].

## 10. Fate-Relevant Endpoints for PLA Biodegradation

Comparisons between glucose, PLA, cellulose, and lignin require standardized incubation conditions because soil type, moisture, temperature, and particle size strongly alter rates and mechanisms. Controlled designs help isolate substrate effects from environmental variation, especially because PLA biodegradation readily occurs at thermophilic temperatures (>50 °C) and proceeds far more slowly under typical mesophilic soil conditions, which can otherwise confound cross-substrate comparisons [14,18,21]. Endpoints should be selected to capture both polymer transformation and carbon fate, since disintegration and molecular weight loss can occur without substantial mineralization (Table 2).

Throughout this review, we use transformation as the broad term for physical or chemical change, depolymerization for molecular weight reduction, biodegradation for biologically mediated conversion that includes assimilation and mineralization, and mineralization for conversion to CO_2_ or CH_4_. There are multiple approaches for quantifying these processes, but not all are equally informative for comparing complex polymers to biological substrates. CO_2_ evolution is the most common metric (e.g., employed in standardized soil biodegradability tests such as ASTM D5988 [80]) because it is easy to measure and indicates mineralization when proper controls are performed. However, CO_2_ can be misleading when SOM priming contributes substantially to respiration or when a significant fraction of a plastic’s carbon is microbially assimilated. Moreover, CO_2_ evolution alone does not resolve whether carbon is transiently incorporated into microbial biomass, retained as microbial necromass, or stabilized as MAOM. For polymers like PLA, which commonly undergo photochemical pre-conditioning at the surface (e.g., Norrish) along with hydrolysis, enzymatic depolymerization, and microbial uptake, CO_2_ endpoints can underrepresent early transformation while overrepresenting priming in active soils, and differences in substrate lability can yield strongly divergent CO_2_ release profiles even in identical soils [67]. Where radiolabeled material is available, ^14^C-based mineralization assays can distinguish substrate-derived ^14^CO_2_ from background respiration and support mass balances among mineralized, residual polymer, and soil-associated pools. This xenobiotic fate approach has now been applied directly to ^14^C-labeled PLA in soil [81].

Polymer transformation metrics also give complementary information. Molecular weight decline (e.g., gel permeation chromatography/GPC) indicates chain scission and can capture early abiotic and biotic depolymerization even when mineralization remains low. Changes in crystallinity, glass transition, tensile properties, and thermal properties (e.g., Thermogravimetric Analysis/TGA) can reflect shifts in polymer accessibility and susceptibility to enzymatic attack. Surface area loss, embrittlement, and tensile property decline are relevant for field exposures and film disintegration. However, these polymer transformation metrics are not equivalent to biodegradation or mineralization because fragmentation and oxidation can be abiotic in nature [82].

Resolving these pools requires analytical methods matched to the size range of the products. Size exclusion or gel permeation chromatography characterizes residual polymer and higher oligomers and is the standard route to M_n_, M_w_, and dispersity, but it requires dissolution in a compatible solvent, calibration against narrow standards, and recognition that material extracted from soil can carry co-eluting organic matter [20,29]. Low-molecular-weight oligomers fall below the reliable range of most size exclusion calibrations and are better resolved by liquid chromatography–mass spectrometry or by matrix-assisted laser desorption/ionization time-of-flight mass spectrometry, which separate individual oligomer series and can distinguish linear from cyclic species. Lactate itself is quantified by high-performance liquid chromatography or ion chromatography alongside other low-molecular-weight organic acids, with the caveat that soil solution concentrations are typically low and turnover is rapid [62,63,64]. Nuclear magnetic resonance spectroscopy provides structural and stereochemical confirmation where sample quantity permits. For PLA specifically, in situ depolymerization coupled to liquid chromatography–tandem mass spectrometry allows polymer mass in an environmental matrix to be quantified as released lactic acid, offering a polymer-specific alternative to spectroscopic particle counting [83]. Extraction efficiency, solvent compatibility, filtration cut-offs, calibration range, and limits of detection should be reported in each case because the apparent absence of soluble products is otherwise indistinguishable from failure to recover them.

To evaluate soil-relevant fate, studies should include carbon partitioning endpoints where feasible. Dissolved organic carbon, water-soluble oligomers, or lactate can indicate hydrolysis products available to the community, but these pools may remain low in situ because of rapid microbial uptake. Mineral adsorption is another plausible explanation, but direct PLA-specific evidence for sorption of released lactate or oligomers is presently insufficient. Physical fractionation approaches that separate POM and MAOM can resolve whether substrate-derived carbon is retained in protected pools, particularly when combined with isotopic labeling of PLA. This is important because microbially processed inputs are predicted to contribute disproportionately to MAOM formation and longer-lived soil carbon, while less processed residues tend to persist as particulate pools [16]. Measuring microbial biomass incorporation and necromass formation provides a mechanistic link between depolymerization, assimilation, and stabilization [16].

When CO_2_ or bulk DOC cannot resolve substrate-derived fate, isotopic attribution approaches can directly link carbon assimilation to taxa and pools. Stable isotope labeling paired with PLFA, amino sugar, or DNA-based methods can identify guilds that incorporate substrate-derived carbon and distinguish assimilation from priming effects. In particular, DNA stable isotope probing provides a direct route to identifying the organisms that incorporate labeled substrate carbon into DNA [84], and multisubstrate DNA-SIP experiments show that bioavailability structures the guilds that assimilate carbon and helps explain differences in mineralization dynamics across substrates [67]. These tools are not required for all studies, but they provide the clearest route to separating PLA-derived carbon from native soil carbon and identifying the organisms and pools responsible for retention versus loss.

## 11. Ecotoxic Effects of Slow and Incomplete PLA Biodegradation

The slow and incomplete transformation of PLA, which can yield persistent microplastic fragments and may generate smaller nanoscale particles as weathering continues, has major implications for agriculture and plant health. Direct evidence for PLA nanoplastic formation under environmentally realistic conditions remains limited. There is a substantial literature on the many negative impacts of microplastics in general on plant health [85]. These impacts include oxidative stress, genotoxicity, cytological abnormalities, disruption of photosynthesis and nutrient uptake, and alteration of gene expression. Microplastics can also serve as vectors for heavy metals and pathogens. However, the vast majority of these studies have focused on traditional non-biodegradable petroleum-based plastics. With the rapid growth of biodegradable plastics, there has been a small and quickly emerging literature documenting the effects of PLA on plant health. Most of these effects are based on experimental lab treatments and affect a diverse range of crop plants in different ways. Examples include reduced root length in soybean [86], reduced root/shoot biomass and chlorophyll in corn [87], growth inhibition and physiological/oxidative stress in buckwheat [88], major reductions in biomass, chlorophyll, and photosynthetic activity in rice [89], reduced shoot weight in Chinese cabbage [90], and impaired growth and disruption of root-exudate/rhizosphere interactions in maize [91]. In general, studies show a dose–response relationship whereby harmful effects increasingly occur at higher PLA concentrations (e.g., [87]). Low concentrations sometimes show little or no effect on plant growth [92] and may even have positive impacts such as increased growth and germination rate in corn at very low doses [93].

Importantly, studies documenting harmful impacts of PLA on plant health often also provide information on the physical, chemical, and biological mechanisms causing those effects. For example, reduced root/shoot biomass and chlorophyll have been attributed to PLA microplastic-induced changes in soil physical properties, microbial community structure, and enzyme activities involved in carbon and nitrogen cycling [87]. Similarly, Lin et al. [94] found that PLA particles reduced rice growth and biomass by disrupting nutrient uptake, increasing oxidative stress, altering antioxidant enzyme activity, changing rhizosphere bacterial composition, and disrupting soil nitrogen and phosphorus cycling. Their results also implicated lactic-acid-derived transformation products, suggesting that toxicity may arise from both the physical presence of particles and products generated during PLA transformation. Finally, a full-crop-cycle study found that PLA microplastics altered carbon, nitrogen, and phosphorus cycling and reduced soil ecosystem multifunctionality over time [91]. While plant responses may vary by species and exposure level, these long-term disruptions can constrain crop growth and health.

Effects attributable to PLA transformation products must be separated from those of the particles themselves and from formulation-derived leachates, and three sources of bioactivity are conflated in much of the current literature. First, lactate is a common microbial metabolite, but accumulation of lactate and soluble PLA oligomers can acidify the immediate microenvironment. Therefore, localized pH depression, rather than molecular toxicity, may account for some reported effects, with dose and buffering capacity determining whether release is inert or inhibitory. Second, oligomers can behave differently from the monomer. Enzymatic hydrolysis of PLA was shown to release oligomers that self-assemble into nanoparticles and provoke inflammatory responses in a mammalian model, indicating that partially depolymerized material can exhibit bioactivity [95]. Third, commercial PLA products contain additives, residual catalysts, and processing aids; in vitro screening of bioplastic and plant-based products found that the majority of samples induced baseline toxicity, and a PLA film was among those showing endocrine activity [96]. Because most soil studies expose organisms to formulated materials rather than purified polymers, effects reported for PLA microplastics cannot at present be assigned to the polymer, its degradation products, or its additives without leachate characterization and appropriately designed controls.

## 12. Engineered Degradation Routes and Practical Feasibility

While our focus in this review is primarily on PLA biodegradation under mesophilic soil conditions, we must also examine the engineered pathways of PLA degradation. Chemical recycling exploits the same ester linkage that governs environmental hydrolysis. Hydrolysis at elevated temperatures, with acid or base catalysis, returns PLA to lactic acid that can be repolymerized. Alcoholysis, most commonly methanolysis or ethanolysis, yields alkyl lactates using metal, organocatalytic, or ionic liquid systems, while glycolysis and aminolysis give diol- and amide-functionalized products for other applications. Thermal depolymerization regenerates lactide directly, closing the loop to the monomer without passing through the free acid [97]. Preserving stereochemistry is key across all of these routes because racemization degrades the value of the recovered monomer [97].

Feasibility at scale is limited less by the chemistry than by the state of the feedstock. Mechanical recycling works for clean, dry PLA products of known composition. Additionally, molecular weight falls substantially over repeated extrusion cycles, with post-consumer material degrading more severely than pre-consumer scrap because of prior aging, washing, and hydrolysis [98]. Chemical routes tolerate more heterogeneity but remain sensitive to contamination, additives, and racemization, and all of them require sufficiently pure and concentrated input to be economically viable [98]. PLA occurs at low concentrations in mixed waste, so collection and sorting of PLA is usually the rate-limiting step [98,99]. Enzymatic depolymerization is an interesting alternative because it operates at lower temperatures with high selectivity, but reaction rates, enzyme stability, and enzyme recovery remain barriers to scale, and recovering lactic acid from dilute aqueous product streams has its own separation and energy costs [98].

These engineered methods and their unique constraints reinforce the central argument of our review. Both industrial composting and chemical recycling can achieve rapid and near-complete conversion of PLA, and both do so by supplying conditions, particularly sustained elevated temperature, controlled moisture, catalysis, or purified feedstock, that ambient soils do not provide. Certification obtained under those conditions describes performance in a managed system, not fate in a field. Functioning collection, reliable end-of-life labeling, and supporting regulatory and economic incentives are key prerequisites for the designed circularity of PLA to be realized [99], and without them, the environmental pathways we describe remain important.

## 13. Future Perspectives

Progress on PLA environmental fate requires evidence at the scales where the uncertainty actually lies, and we see eight priorities. First, conducting field-relevant mesocosm and multiseason experiments under ambient temperature and moisture, since most mechanistic understanding derives from short incubations under controlled conditions. Second, determining complete carbon mass balances for isotopically labeled PLA that partition polymer-derived carbon among CO_2_, microbial biomass, dissolved organic carbon, particulate organic matter, and mineral-associated organic matter, rather than reporting mineralization alone. Third, verifying depolymerizing taxa and cross-feeding using stable isotope probing and community-resolved methods to test the trait-gating framework advanced here. Fourth, measuring molecular weight and soluble products alongside CO_2_ so that transformation is not inferred from disintegration. Fifth, promoting formulation and additive transparency for commercial products so we can attribute observed effects to the polymer. Sixth, developing PLA-specific detection methods for micro- and nanoplastics able to distinguish PLA from total microplastic loads in soils, biosolids, and waters. Seventh, testing ecotoxicity at environmentally defensible concentrations, with leachate controls that separate particle, degradation product, and additive effects. Eighth, evaluating economics of collection, sorting, and depolymerization pathways at the scale that PLA end-of-life claims presuppose. Addressing these would convert contextual recalcitrance from an organizing framework to a quantitative and predictive account of PLA fate.

## 14. Conclusions

The environmental fate of polylactic acid, a biobased and biodegradable alternative to conventional plastics, is strongly context-dependent on polymer and microbial community characteristics and cannot be inferred from compostability certifications alone. We propose contextual recalcitrance as a framework for explaining why PLA can satisfy industrial compostability criteria yet persist in ambient soils and waters. PLA transformation is constrained by polymer physicochemical structure and environmental controls on hydrolysis and enzyme activity, mainly under mesophilic conditions where depolymerization can be slow. As a result, fragmentation, molecular weight loss, and partial surface erosion can occur without rapid mineralization, and outcomes vary widely across sites and experimental designs.

Consequently, the rapidly growing literature documents that “biodegradable” PLA materials used in agriculture can transform slowly and incompletely and can generate persistent microplastic fragments that negatively impact plant health via a variety of physical, chemical, and biological mechanisms. The generation of PLA nanoplastics is also plausible as fragmentation continues, although their abundance and effects under environmentally realistic conditions remain insufficiently quantified. Indeed, one recent paper referred to PLA products as a “biohazard” [94]. This realization underscores the importance of understanding the specific processes occurring during PLA degradation. Polylactic acid degradation is likely gated by a limited set of microbial traits associated with extracellular depolymerization and colonization, while community-level carbon fate may be influenced by competition and cross-feeding of lactate and oligomers. This implies that the term “PLA degraders” is not sufficient for predicting carbon outcomes. Instead, the key question is how quickly depolymerization generates bioavailable products, how those products are allocated among community members, and whether resulting biomass and necromass are retained in relatively more protected soil organic matter pools such as MAOM. Ideally, this understanding will support formulations and end-of-life systems that degrade more predictably and produce fewer harmful impacts.

## Figures and Tables

**Figure 1:**
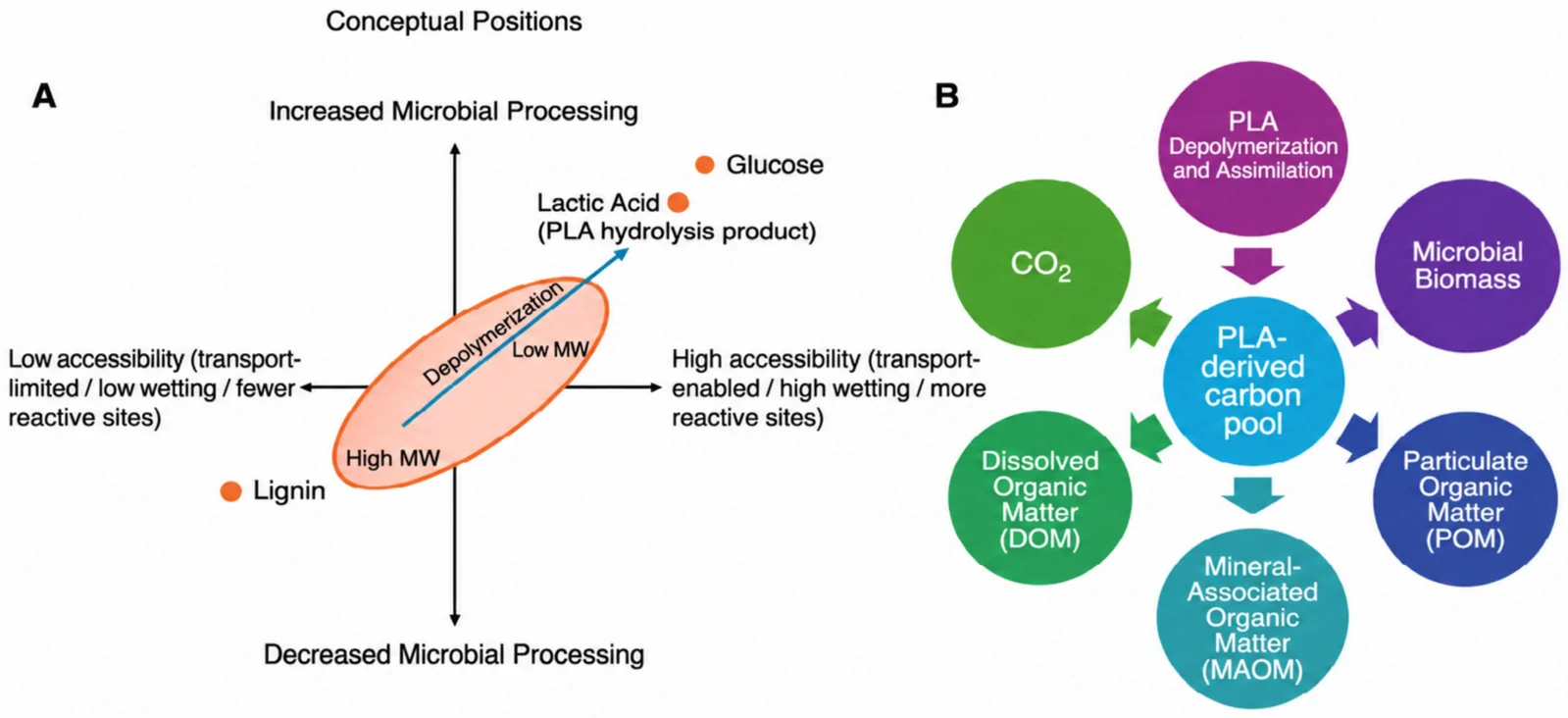
Conceptual framework for polylactic acid (PLA) transformation and carbon fate in soil. Positions, arrows, and relative spacing are qualitative and conceptual; they are not based on measured values and do not represent rates, distances, effect sizes, or flux proportions. (**A**) PLA and reference substrates are positioned within an accessibility–processing space. The x-axis represents effective physicochemical accessibility, including wetting, diffusion, and reactive surface area, while the y-axis represents the degree of microbial processing, from limited transformation to rapid uptake and turnover. High-molecular-weight PLA (High MW) occupies a context-dependent conceptual position and becomes more accessible as depolymerization lowers molecular weight (Low MW). (**B**) Following depolymerization, PLA-derived carbon may be mineralized to CO_2_, assimilated into microbial biomass, released as dissolved organic matter (DOM), retained as particulate organic matter (POM), or potentially associated with mineral-associated organic matter (MAOM). Actual partitioning depends on environmental conditions, microbial traits, and soil mineralogy.

**Figure 2:**
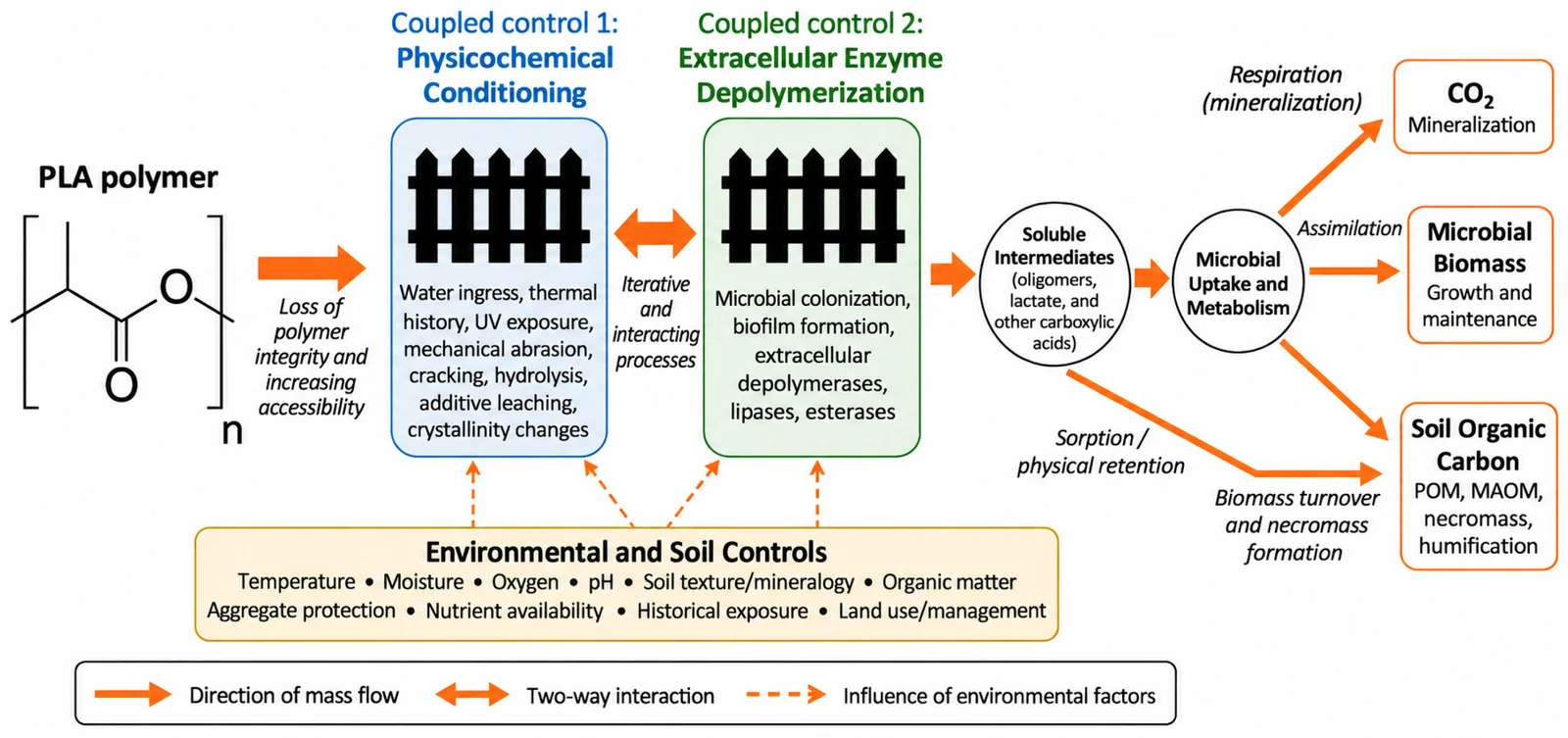
Coupled physicochemical and biological controls on PLA depolymerization and carbon fate in soil. Physicochemical conditioning, including water ingress, thermal and photochemical weathering, and surface cracking, interacts iteratively with microbial colonization and extracellular enzyme activity to generate soluble oligomers and lactate. These products may be mineralized to CO_2_, incorporated into microbial biomass, or potentially retained in soil as other forms of organic carbon (particulate organic matter (POM), mineral-associated organic matter (MAOM), necromass) through hypothetical sorption and microbial residue formation.

**Table 1:** Evidence map for PLA depolymerization mechanisms and trait gating from enzyme to isolate to soil incubations. Evidence categories run from purified enzyme/biochemical to isolate and laboratory microcosm to mesocosm and field, with indirect ecological analogy as the weakest tier; no field-scale study currently resolves PLA depolymerization mechanisms, and that gap is itself informative.

Mechanism/Trait Focus	Representative Taxa/System	Evidence Type	Key Conditions (Temperature, Time, Medium)	Key Citation	Evidence Category and Level of Environmental Inference
Extracellular depolymerase as the rate-limiting gate (primary depolymerization)	*Amycolatopsis* sp. strain K104-1 (soil isolate)	Purified enzyme + growth/assimilation assays	Basal mineral medium with 0.1% (*w*/*v*) emulsified PLA in 10.7 mM potassium phosphate buffer, pH 7.1; Growth and assimilation at 37 °C for up to 8 d; culture supernatant collected after 5–7 d for enzyme purification; purified enzyme optimum pH 9.5 and 55–60 °C	Nakamura et al. [31]	Purified enzyme/biochemical—direct for enzyme capability; indirect for environmental communities
Trait rarity and phylogenetic clustering of primary PLA degraders	*Actinomycete* culture collections (41 genera; 105 strains)	Screening survey (clear zone on emulsified PLA)	PLA-emulsified agar screening (1 g L^−1^ PLA), 30 °C; PLA film and liquid culture follow-up for 14 d	Jarerat et al. [32]	Isolate (screening survey)—direct for the tested collection; indirect for field prevalence
Biofilm formation and surface colonization as a prerequisite for effective film deterioration	*Amycolatopsis* isolates from soil (strain SNC highlighted)	Isolates + film biodeterioration tests + microscopy	Mesophilic; 1 month film incubation (media included soil extract medium; gelatin used as inducer in some treatments)	Decorosi et al. [33]	Isolate—direct strain-level colonization and film deterioration; indirect for communities
Screening-to-verification attrition (many positives on emulsions, few on solid polymer)	*Amycolatopsis* isolates from soil (strain SNC highlighted)	Methodological comparison embedded in primary study	0.1% (*w*/*v*) emulsified PLA in basal medium with or without 0.1% gelatin; 30 °C, 100 rpm, 7 d; solid film verification in soil extract medium for 30 d	Decorosi et al. [33]	Isolate (method comparison)—direct evidence of screening attrition; indirect for environmental prevalence
Inoculum limitation/degrader scarcity (bioaugmentation effect)	*Pseudonocardia* sp. RM423 added to soil	Soil microcosms: bioaugmentation vs. control	Compared 30 °C vs. 58 °C; 60 d (soil assays in study; additional submerged assays reported)	Apinya et al. [21]	Laboratory microcosm—direct controlled evidence; moderate environmental inference
Biostimulation vs. bioaugmentation (enzyme investment limits baseline mineralization)	Soil microcosms (natural attenuation vs. bioaugmentation vs. biostimulation)	Soil incubation experiments	30 °C; 150 d	Satti et al. [24]	Laboratory microcosm—direct controlled evidence; moderate environmental inference
Surface-mediated enzyme kinetics (accessibility controls rates)	Proteinase K (model depolymerase)	In vitro surface erosion kinetics	Enzyme concentration series; erosion rate plateau at >100 µg/mL proteinase K	Yamashita et al. [34]	Purified enzyme/biochemical—direct surface kinetics with a model enzyme; indirect for intact soil systems
Multiple enzyme classes can depolymerize PLA, but solid-surface constraints matter	Microbial carboxylesterases (metagenome-derived; biochemical/structural characterization)	Enzyme biochemistry + structural biology	Lab assays; structural characterization	Hajighasemi et al. [35]	Purified enzyme/biochemical—direct enzyme capability; indirect for intact environmental communities
CO_2_-only endpoints can misestimate biodegradability (priming confound; assimilation vs. mineralization)	Agricultural soils incubated with PLA microplastics	Soil incubations with isotopic attribution + biomarker tracing	Multiple soils; incubation study	Yu et al. [36]	Laboratory microcosm with isotope attribution—direct evidence of PLA-derived carbon fate; strongest environmental inference in this table

**Table 2:** Definitions and endpoints for PLA degradation.

Endpoint	Definition	Example Measurements
Polymer transformation: fragmentation	Physical breakup, does not imply chemical or biological conversion	Film thinning, mass loss, particle counts
Polymer transformation: depolymerization	Reduction in molecular weight via hydrolysis/photochemistry and/or enzyme-mediated chain scission, yielding oligomers/monomers	M_w_/M_n_ (GPC), FTIR changes
Biodegradation (umbrella process; follows or accompanies depolymerization)	Biologically mediated conversion following depolymerization; includes assimilation and mineralization	PLA-derived C incorporated into biomass and/or recovered as CO_2_/CH_4_; isotope attribution when feasible
Mineralization (biodegradation outcome)	Fraction converted to CO_2_/CH_4_	Radio- or stable isotope tracing
Assimilation (biodegradation outcome)	Incorporation into microbial biomass	Biomass, PLFA/DNA-SIP, amino sugars
Subsequent environmental fate: stabilization, retention, or redistribution	Persistence in stable carbon pools versus loss as mineralization	Density fractionation, MAOM/POM partitioning

Abbreviations: DNA-SIP, DNA stable isotope probing; FTIR, Fourier transform infrared spectroscopy; GPC, gel permeation chromatography; MAOM, mineral-associated organic matter; M_n_, number-average molecular weight; M_w_, weight-average molecular weight; PLFA, phospholipid fatty acid; POM, particulate organic matter.

## Data Availability

No new data were created or analyzed in this study. Data sharing is not applicable to this article.

## Supplementary Materials

The following supporting information can be downloaded at https://www.mdpi.com/article/10.3390/jox16060183/s1: Table S1: Representative studies testing PLA degradation and related biodegradable plastic benchmarks in environmental systems [18,19,20,21,22,23,24,25,26,27,28,29,30].
