# Supplementary material for: Contextual Recalcitrance of Polylactic Acid (PLA) in Soils: Degradation Mechanisms, Microbial Traits, and Fate-Relevant Endpoints
Source: J Xenobiot. 2026 Sep 25;16(6):183. doi: 10.3390/jox16060183 (PMC13705997; doi:10.3390/jox16060183)
Supplement: Supplementary file 1 [file jox-16-00183-s001.zip › jox-4543852-supplementary.pdf]

Supplementary materials: Contextual Recalcitrance of Polylactic Acid (PLA) in Soils: Degradation Mechanisms, Microbial Traits, and Fate-Relevant Endpoints

Jacob W. Clements, Michael L. McKinney, Jennifer DeBruyn, Douglas G. Hayes and Sean M. Schaeffer

Table S1. Representative studies testing PLA degradation and related biodegradable-plastic benchmarks in environmental systems

Study	Material and composition	System	Conditions	Primary endpoints	Key findings and environmental relevance
**Ho & Pometto (1999) [18]**	Commercial PLA films (multiple types)	Laboratory soil respirometers	28, 40, and 55 °C; 182 d	Cumulative CO_2 mineralization	Strong temperature dependence: approximately 27%, 45%, and 70% mineralization at 28, 40, and 55 °C, respectively, after 182 d.
**Ho et al. (1999) [19]**	PLA films (Chronopol and Cargill Dow types)	Field soil and compost rows	Banana-field soil, Costa Rica (approximately 27 °C; high moisture); compost rows, Iowa (55 ± 5 °C; 50 ± 10% relative humidity)	Molecular-weight decline (Mw per week)	Molecular-weight loss indicated degradation on a weeks timescale in compost rows versus a months timescale in tropical soil.
**Saadi et al. (2012) [20]**	Poly(L-lactide) (PLLA) films; fungal inoculation was a treatment, not a polymer blend	Sterilized agricultural soil and compost, with or without a fungal consortium	Soil: 30 °C, 220 d. Compost: 30 or 58 °C, up to 230 d; moisture maintained at 80–100% of water-holding capacity	CO_2 mineralization; M_n; glass-transition temperature; spectroscopy and morphology	In fungal-inoculated soil at 30 °C, no CO_2 was detected for 19 d and biodegradation reached 8% by day 220; the cellulose reference reached 84% by day 75. In fungal-inoculated compost at 58 °C, biodegradation reached approximately 90% by day 230. M_n declined from 116,000 to 2,100 g mol^{-1} by day 38 and 780 g mol^{-1} by day 70; glass-transition temperature declined from 60.8 to 43.8 °C by day 70.
**Apinya et al. (2015) [21]**	PLA sheets; bioaugmentation with Pseudonocardia sp. RM423	Soil microcosms	30 °C or 58 ± 2 °C; 60 d; bioaugmented and uninoculated treatments	Weight loss and CO_2 evolution	RM423 bioaugmentation produced 74.6% PLA weight loss at 58 °C versus 0.9% at 30 °C after 60 d, showing that thermophilic conditions plus an effective degrader can shift PLA from near-stable to rapid mass loss.
**Muniyasamy et al. (2016) [22]**	Neat PLA, PHBV, and a PLA/PHBV blend	Composting and soil burial	200 d total	CO_2 mineralization; FTIR, SEM, and TGA	PLA reached approximately 90% biodegradation during composting but only approximately 4% during soil burial over 200 d.
**Palsikowski et al. (2018) [23]**	Neat PLA, neat PBAT, and PLA/PBAT blends (75/25 and 25/75, w/w) compatibilized with 1 phr Joncryl ADR-4368	Controlled laboratory soil burial	360 d; periodic retrieval	Visual change; SEC; DSC; FTIR; respirometry	After 360 d, PBAT remained as small fragments, both blends fragmented, and PLA mainly cracked. Mineralization ranked PBAT > 25/75 blend > 75/25 blend ≈ PLA; chain extension slowed molecular-weight and carbonyl-index changes.
**Satti et al. (2018) [24]**	PLA films; treatments differed in microorganisms and nutrients, not polymer formulation	Soil microcosms: natural attenuation, bioaugmentation, and biostimulation	30 °C; 150 d; abiotic controls included	CO_2 mineralization	Natural mineralization was slow (approximately 10% by 150 d). Bioaugmentation and biostimulation increased mineralization to approximately 22–24% by 150 d.
**Sintim et al. (2020) [25]**	Commercial soil-biodegradable mulch films; formulations varied and were partly proprietary, and PLA content was not confirmed for every film	Compost and agricultural field soil at warm and cool sites	Compost: 18 weeks. Soil: 36 months; warm-climate Knoxville and cool-climate Mount Vernon	Surface-area reduction; FTIR; molecular-weight change; TGA; NMR	Surface-area reduction reached 85–99% in compost after 18 weeks and 61–83% (Knoxville) or 26–63% (Mount Vernon) in soil after 36 months. This study is included as a real-world biodegradable-film benchmark; its rates should not be attributed specifically to neat PLA.
**Slezak et al. (2023) [26]**	PLA1: PLA with PBAT and additives; PLA2: PLA-based polyester blend with mineral filler	Field soil under natural conditions	One year; mean annual temperature 9.4 °C; winter and summer exposure	Mass loss; macro- and microscopic observations; thermal and tensile tests	PLA1 and PLA2 lost <0.6% mass after 12 months. Surface erosion and shifts in tensile and thermal properties occurred despite minimal mass loss.
**Royer et al. (2023) [27]**	100% PLA textile fibers; PET, PP, and a 50/50 cellulose–PET blend were comparators	Coastal seawater exposures (surface and seafloor) plus a 28 d ASTM D6691 marine bioreactor	Field: 231 d at the surface and 196 d at the seafloor (14–24 °C); flow-through aquarium extension to 428 d	Disintegration and visual monitoring; Raman spectroscopy; bioreactor CO_2 mineralization	PLA did not disintegrate during 196–231 d in situ and remained intact after the 428 d extension. Raman spectra were unchanged at day 84, indicating no detectable chemical weathering at that time.

**Poli et al. (2025) [28]**	PLA pellets	Aerobic freshwater biodegradability test (EN ISO 14851:2019) using two inoculum sources	20–25 °C; incubation continued until biochemical oxygen demand plateaued	Biochemical oxygen demand; pellet mass; FTIR, DSC, and SEM	Reported biodegradation was 44.04% with inoculum α and 23.38% with inoculum β. No measurable pellet mass change occurred, and FTIR, DSC, and SEM showed negligible structural change.
**Szewc et al. (2025) [29]**	PLA microplastics; polystyrene microplastics were comparators	Marine water and oxygen-deficient fine-grained marine sediment	3 months; marine water at 6–8 °C; oxygen-deficient sediment; low-temperature incubation	SEC/GPC (M_n and PDI); SEM; gravimetric mass loss	In marine water, combined biotic and abiotic hydrolysis reduced M_n by 17% in 3 months, while mass loss was only 0.02–0.08%. No molecular-level degradation was detected in oxygen-deficient fine sediment, indicating persistence under cold, low-oxygen benthic conditions.
**Chen et al. (2024) [30]**	PLA films (0.25 ± 0.05 mm); PGA and PLGA films were comparators	Simulated marine environment (artificial seawater and marine sediment; approximately 34 PSU; aerobic interface)	Up to 300 d; major visible change in PLA after approximately 120 d	FTIR and XPS; GPC; DSC and XRD; TGA; SEM; visual fragmentation	PLA showed delayed transformation, with visible embrittlement and fragmentation after approximately 120 d. The XPS C–O/–COO– peak-area ratio increased from 1.30 to 2.22 after 300 d, and M_n declined over time more slowly than for PGA and PLGA.

Note: Abbreviations: CO_2, carbon dioxide; d, days; DSC, differential scanning calorimetry; FTIR, Fourier-transform infrared spectroscopy; GPC/SEC, gel/size-exclusion chromatography; M_n, number-average molecular weight; Mw, weight-average molecular weight; NMR, nuclear magnetic resonance; PBAT, poly(butylene adipate-co-terephthalate); PDI, dispersity; PET, polyethylene terephthalate; PGA, poly(glycolic acid); PHBV, poly(3-hydroxybutyrate-co-3-hydroxyvalerate); phr, parts per hundred resin; PLA, poly(lactic acid); PLGA, poly(lactic-co-glycolic acid); PP, polypropylene; PSU, practical salinity units; SEM, scanning electron microscopy; TGA, thermogravimetric analysis; w/w, weight/weight; XPS, X-ray photoelectron spectroscopy; XRD, X-ray diffraction. Percent values retain the endpoint definitions used by the cited studies and therefore are not necessarily directly comparable among studies.
